# Postharvest Behavior of Two Peach Varieties During Cold Storage and Shelf Life: Physiological and Sensory Perspectives

**DOI:** 10.3390/plants14213291

**Published:** 2025-10-28

**Authors:** Víctor David González, Leandro Arrillaga, Olga Pascual, Fernanda Zaccari, Ana Cecilia Silveira

**Affiliations:** Poscosecha de Frutas y Hortalizas, Departamento de Producción Vegetal, Facultad de Agronomía, Universidad de la República (Udelar), Avda. Garzón 780, Montevideo CP 12900, Uruguayfzaccari@fagro.edu.uy (F.Z.)

**Keywords:** peach, fruit quality, firmness, total phenolics, antioxidant capacity, sensory evaluation, shelf life

## Abstract

The postharvest behavior of two peach varieties (Pavía Canario and Moscato Tardío) was evaluated during 30 days of refrigerated storage at 0 °C followed by 5 days at 20 °C (shelf life). Firmness, respiratory activity, sugar content, total polyphenol content (TPC), total antioxidant capacity (TAC; ferric reducing antioxidant power, FRAP; 2,2-diphenyl-1-picrylhydrazyl, DPPH; and 2,2′-azino-bis (3-ethylbenzothiazoline-6-sulfonic acid), ABTS), and sensory attributes were assayed. Respiratory activity peaked around day 15 in both varieties, with Moscato Tardío showing higher values than Pavía Canario, reflecting differences in cold stress response. During refrigerated storage, Moscato Tardío maintained higher firmness (36.1 N) than Pavía Canario (27.2 N), although firmness decreased by ~30% after transfer to shelf life. Glucose and sucrose contents were higher in Moscato Tardío, while Pavía Canario showed higher initial TAC (FRAP: 117.2 mg ascorbic acid equivalents AAE·kg^−1^; DPPH: 472.8 mg AAE·kg^−1^). TPC increased over time in both varieties, but with different dynamics: Pavía Canario peaked at 20 days, whereas Moscato Tardío increased markedly at 30 days. ABTS values varied intra-varietally, showing maximum differences during storage. Sensory evaluation indicated better juiciness and reduced mealiness in Moscato Tardío throughout storage and shelf life. Overall, postharvest responses were genotype-dependent: Pavía Canario activated antioxidant defenses earlier, while Moscato Tardío exhibited delayed but substantial responses, maintaining firmness and sugar content, and better overall sensory quality.

## 1. Introduction

The peach (*Prunus persica* (L.) Batsch) originated in China around 1100 BC. It is a widely known and consumed fruit worldwide, ranking third among commercially important fruits after apple and pear [1,2]. In turn, it is one of the species that presents the most significant variability in terms of shape, size, color of pulp and peel (red, yellow, white), and other aspects, which determines a great variety of commercial cultivars [3,4]. In addition to their exotic flavor, the fruits are appreciated for being rich in compounds of nutritional and functional interest, such as carbohydrates, acids, amino acids, and compounds with antioxidant activity, such as polyphenols (chlorogenic acid, catechin, quercetin, cyanidin, among others) and vitamins (A, B, C), carotenoids, among others [5,6]. They are climacteric and very perishable fruits, with a storage time that varies according to the genetic material but usually not exceeding 40 days [7]. The main deterioration processes occur due to the loss of firmness and dehydration. For this reason, prolonging the shelf life using technologies such as refrigeration is essential. Low temperatures allow for a reduction in the metabolism of plant products, such as respiration and ethylene emission, delaying the ripening process and maintaining quality [8,9].

However, using low temperatures for their conservation does not always meet the objective of reducing losses, since many fruits, such as peaches, are sensitive to cold. In cold-sensitive products, exposure to low temperatures causes alterations in metabolism due to an increase in reactive oxygen species (ROS), which act as signaling molecules. ROS first affect cell membrane integrity, causing ion leakage from the cell and the alteration of the electrolyte balance. The variation in ion content affects cellular functions and, consequently, physiological processes [10,11]. These damages manifest as internal browning, decay, loss of ripening capacity, loss of flavor, lack of juiciness (mealiness/woolliness), and, finally, reddish flesh discoloration (reddening/bleeding), among others, which detract from the organoleptic quality and determine the rejection of consumers [12].

Vegetable products possess a natural antioxidant defense system composed of enzymatic and non-enzymatic components, including polyphenols, ascorbic acid, and flavonoids. The high antioxidant activity of these compounds is mainly due to their reducing capacity, which helps protect cellular structures from oxidation. In most cases, chilling injury becomes visible only during post-storage ripening after prolonged cold storage damage [13,14]. There are differences between genetic materials in terms of metabolic activity (respiratory rate and ethylene emission), softening speed, and composition. These differences affect the storage potential as well as the susceptibility to developing chilling injuries, since, according to different authors, susceptibility to chilling is genetically based [15,16]. According to the response of peach fruits to storage at temperatures of 0 or 5 °C for different periods, they can be classified into three categories: not susceptible to chilling and insensitive to temperature; not susceptible to cold, resistant to at least 5 weeks of storage at 0 °C but susceptible to cold (<5 weeks of storage) at 5 °C (temperature sensitive); and, finally, susceptible to cold (<5 weeks of storage) in both storage temperatures [17].

Knowing the ripening process and the behavior of the products during the postharvest period is essential to satisfying consumers and reducing losses. Studies on consumer preferences indicate that peach consumption is decreasing because the fruit maintains a flavorless profile, poor texture, and lower overall quality [18].

The postharvest behavior of two locally bred peach varieties, Moscato Tardío and Pavía Canario, was evaluated by assessing metabolic, compositional, functional, and sensory attributes during cold storage and shelf life. Data on local cultivars remain scarce, and this work provides a comprehensive assessment of their quality changes, offering valuable information for producers, post-harvest management, and breeding programs aimed at improving fruit quality under regional conditions. This study thus provides a solid and multidisciplinary evaluation of cultivar-dependent differences in storage potential and susceptibility to chilling injury.

## 2. Results and Discussion

### 2.1. Respiration Rate

**Figure 1:**
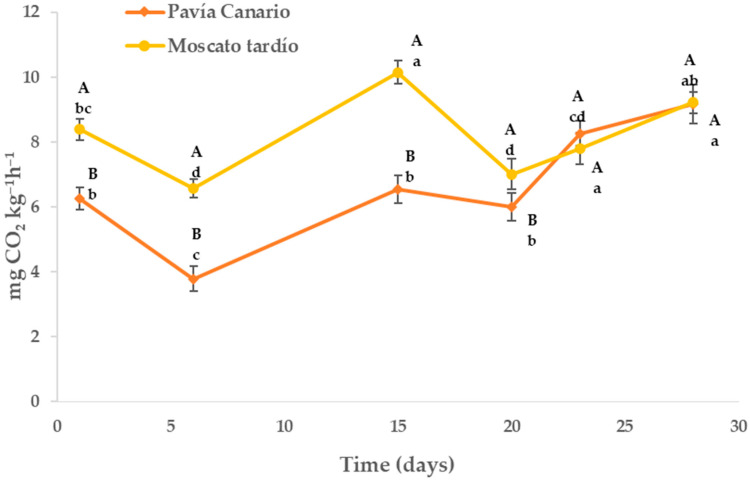
Respiration rate of *Pavía Canario* and *Moscato tardío* peaches during refrigerated storage. Values are means (n = 4) ± standard error of the mean. Uppercase letters indicate differences between varieties at each evaluation day, and lowercase letters indicate differences over time within each variety (Tukey’s test, *p* < 0.05).

In both varieties, respiratory activity decreased after 5 days of storage related to harvest, where the highest value observed corresponds to the stress suffered by the fruits when separated from the plant (Figure 1). After this initial decline, a significant increase was observed, reaching a peak at day 15. This behavior may be related to a new stress situation caused by low temperatures, leading to the onset of chilling injury symptoms. Until 20 days, there was a new decrease in respiration followed by a progressive increase because of the advance of the maturation and senescence process.

On the other hand, the varieties showed differences until 20 days of storage, where Moscato tardío had greater respiration than Pavía Canario. The largest differences were observed at 6 days (42% higher) and the smallest at 20 days (14% higher).

The lower respiratory activity could indicate a greater tolerance to low temperatures since the energy status of the cells is the central point in the resistance to cold of plant products [19,20]. Low efficiency in the respiratory process leads to an energy deficit, promoting the generation of reactive oxygen species (ROS) that cause structural membrane damage, which could explain the physiological disorders resulting from cold damage [21,22].

### 2.2. Flesh Firmness

**Figure 2:**
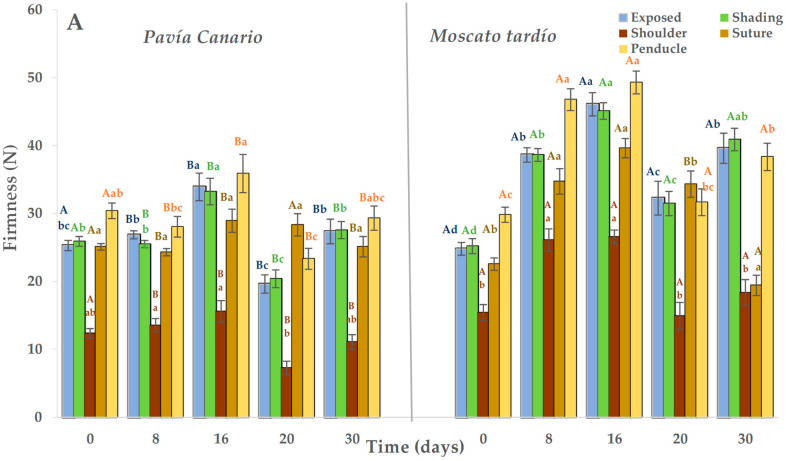

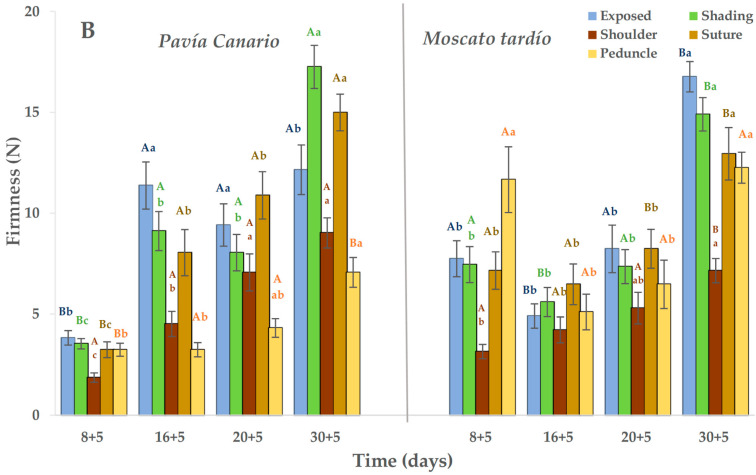
Flesh firmness of Pavía Canario and Moscato tardío peach fruits measured at exposed, shading, shoulder, suture, and peduncle during (**A**) refrigerated storage and (**B**) shelf life. Values are means (n = 21) ± standard error of the mean. Uppercase letters indicate differences between varieties at each evaluation day, and lowercase letters indicate differences over time within each variety (Tukey’s test, *p* < 0.05).

During refrigerated storage, Moscato tardío showed higher firmness than Pavía Canario, with average values of 36.1 N and 27.2 N, respectively. At harvest, no significant differences were observed between varieties in the exposed and shaded sides. However, in the following evaluation periods, Moscato tardío remained approximately 30% firmer than Pavía Canario (Figure 2A). In both varieties, the highest firmness values during storage were recorded at 16 days, exceeding those measured at harvest. In Moscato tardío, the lowest firmness values (in the exposed and shaded sides) were observed at harvest, followed by a progressive increase during cold storage. Among the measured zones, the shoulder exhibited the lowest firmness values in both varieties (20.6 N in Moscato tardío and 12.2 N in Pavía Canario), and both shoulder and suture areas remained relatively stable throughout storage.

Once transferred to the shelf life condition, firmness decreased by approximately 30% in both varieties. Unlike what was observed during cold storage, Pavía Canario was about 20% firmer than Moscato tardío (Figure 2B). The evolution of firmness over time showed that firmness of the exposed side of Pavía Canario increased at 16 + 5 days and was maintained until 30 + 5 days. A progressive increase was also observed on the shaded side, suture, and peduncle, reaching the maximum value at 30 + 5 days. In Moscato tardío, the values measured at 8 + 5 days were higher than those measured at 16 and 20 + 5. Also, the maximum values were reached after 30 + 5 days in all the measured areas.

During the maturation process, it is expected that the firmness will reduce until it reaches values between 9 and 13 N, which correspond to the firmness for consumption, in a period that will depend on the storage temperature. For example, in peaches kept at 20 °C, consumption maturity was reached in 5 days. The unexpected increase in firmness, particularly after transfer to room temperature, could indicate the onset of chilling injury which varies according to genetic background. This behavior is genotype-dependent, as shown in Red Haven peaches, which softened progressively at 0 and 5 °C for 15 days, but after 30 days, fruits at 5 °C retained greater firmness and lost 40% of juiciness [12,23].

In a work where peaches were preserved for different times (1, 2, 3, and 4 weeks) at 0 °C and then placed at 10 °C for 6 days before moving to shelf life conditions (1 day at 20 °C), the loss of firmness was practically minimal in the treatments that were stored for 2, 3 and 4 weeks. The authors attribute this behavior to the low expression of the PpPGM (linked to polygalacturonase enzyme) gene, which plays a crucial role in fruit softening. This enzyme has a strong temperature dependence [24]. In our experiment, it was observed that both varieties were sensitive to cold. However, the evolution of firmness during shelf life could indicate a greater sensitivity of the Pavía Canario variety.

### 2.3. Weight Loss

**Figure 3:**
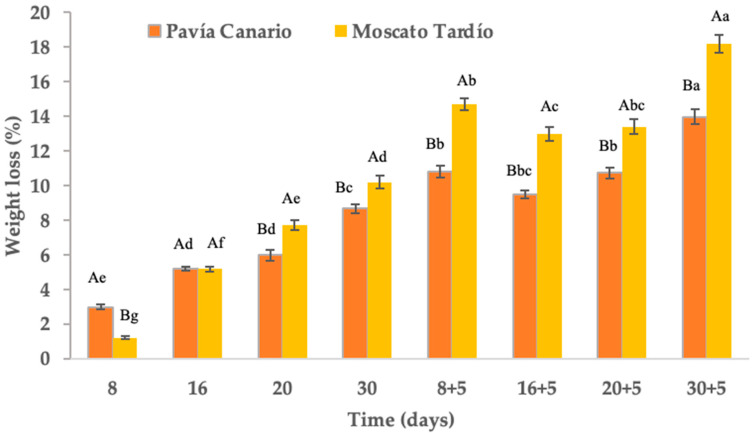
Weight loss of Pavía Canario and Moscato tardío peach fruits Values are means (n = 21) ± standard error of the mean. Uppercase letters indicate differences between varieties at each evaluation day, and lowercase letters indicate differences over time within each variety (Tukey’s test, *p* < 0.05).

After 30 days of refrigerated storage, weight loss reached 8.7% in Pavía Canario and 10.2% in Moscato Tardío (Figure 3). At 8 days of storage, Pavía Canario exhibited greater weight loss, but this trend reversed from 20 days onward when Moscato Tardío showed significantly higher losses. During shelf life, the differences between the genetic materials remained. In many Prunus species, cumulative losses below 10% after 28–30 days at low temperatures are considered acceptable for maintaining marketability [25,26]. Thus, while Pavía Canario remained within acceptable limits, Moscato tardío marginally exceeded them. After 8 days of cold storage followed by 5 days of shelf life, weight loss reached 10.8% in Moscato Tardío and 14.7% in Pavía Canario. At the end of the experiment (30 + 5 days), losses achieved 13.9% and 18.2%, respectively (Figure 3). Both values surpass the 10% commercial limit for fresh peaches, particularly impacting Moscato. This indicates significant dehydration, which is likely to affect texture, visual quality, and consumer acceptance.

According to Gidado et al. [27], water loss in fruits occurs through the cuticle, epidermis, stomata, lenticels, mesocarp, and vascular system. The rate of water loss will depend on the proportion and characteristics of these structures. These aspects have a genetic component that explains the differences observed between varieties, as in this work. The greater initial loss in Pavía Canario may be associated with a thinner cuticle or higher stomatal density, making it more susceptible to dehydration in the early stages. Conversely, the sharper increase in Moscato Tardío at later stages, especially during shelf life, might suggest a delayed onset of dehydration, potentially due to structural or metabolic changes induced by prolonged cold exposure. This could reflect a greater sensitivity to chilling injury, which compromises membrane integrity and accelerates water loss upon transfer to ambient temperatures.

### 2.4. Total Soluble Solid Contents, Sugars and Organic Acid

At harvest, total soluble solid (TSS) was between 11.5 and 13.8 °Brix on the exposed side and between 10.9 and 13.5 °Brix in Pavía Canario and Moscato tardío, respectively. These values remained relatively stable during refrigerated storage and shelf life.

Regarding sucrose, significant differences were found between varieties, with Pavía Canario showing an average content of 2.22 g 100 g^−1^ FW and Moscato tardío 1.98 g 100 g^−1^ FW (Table 1). In Pavía Canario, sucrose content increased after harvest (from 1.81 to 2.74 g 100 g^−1^ at 16 days), followed by a gradual decline until the end of the evaluation (30 + 5 days). In Moscato tardío, sucrose levels decreased slightly during storage and reached their lowest values during shelf life. With respect to glucose and fructose, both were significantly affected by genotype. On average, Moscato tardío showed higher glucose content (1.56 g 100 g^−1^ FW) compared to Pavía Canario (1.20 g 100 g^−1^ FW), while fructose content was more variable between varieties and storage stages. On days 16, 20, and 30, glucose content in Pavía Canario was 20–39% lower than that measured in Moscato tardío, a trend also observed at 8 + 5 days.

Pavía Canario exhibited greater variation in citric acid content during refrigerated storage, while in Moscato tardío, levels decreased steadily, with the highest values at harvest and 8 days, and the lowest at 30 days (Table 1). In shelf life, both varieties showed no significant variation. Significant differences between varieties were observed at harvest, 16 and 30 days, and at 20 + 5 and 30 + 5 days, with Pavía Canario showing nearly double the citric acid content compared to Moscato tardío. The malic acid content in Pavía Canario remained relatively stable until 16 days, then decreased gradually to its lowest level at 30 + 5 days. In Moscato tardío, the highest value was observed at harvest (586.49 mg 100 g^−1^), with no significant differences among subsequent stages. From day 20 onward, Pavía Canario consistently showed higher citric acid levels than Moscato tardío.

Previous studies report that malic acid content in peach fruit was 4–5 times higher than citric acid content, in agreement with previous reports [28,29]. On the other hand, sugars and organic acids are affected by cold storage and may function as osmoregulatory compounds, cryoprotectants, or signaling molecules [16,30]. Sugar content differences between varieties influence not only flavor but also susceptibility to chilling injury. In this sense, Moscato tardío exhibited higher TSS, sucrose, and glucose content, potentially indicating greater cold tolerance. According to Zhao et al. [31] sucrose accumulation is associated with increased cold tolerance when peaches are stored at sub-zero temperatures (−1.4 ± 0.1 °C). Moreover, various studies report that postharvest treatments aimed at reducing chilling injury, such as salicylic acid, glycine, or jasmonic acid, help maintain or even enhance sugar content, especially sucrose. High sucrose content may confer several benefits, including membrane stabilization and ROS scavenging under cold stress conditions [31,32,33,34].

Organic acids have also been linked to chilling injury. Navarro et al. [30] found a positive correlation (r = 0.630 *) between malic acid and internal browning in Big Top peaches grafted on Adarcias rootstock. In the present study, the higher malic acid content in Moscato tardío at harvest could be associated with greater susceptibility to cold damage.

### 2.5. Total Polyphenol Content and Antioxidant Capacity

#### 2.5.1. Total Polyphenol Content

**Figure 4:**
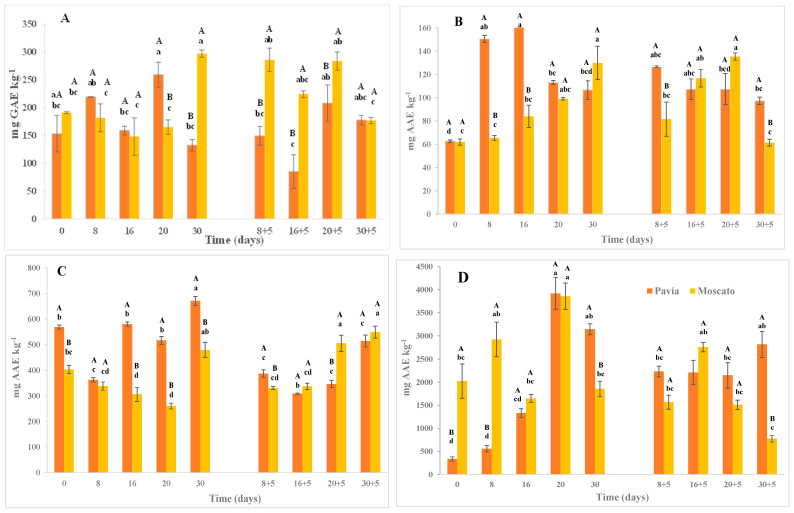
Total polyphenol content (**A**) and antioxidant capacity measured by FRAP (**B**), DPPH (**C**) and ABTS (**D**) of Pavía Canario and Moscato tardío peach fruits. Values are means (n = 3) ± standard error of the mean. Uppercase letters indicate differences between varieties at each evaluation day, and lowercase letters indicate differences over time within each variety (Tukey’s test, *p* < 0.05).

The total polyphenol content (TPC) measured by spectrophotometry showed significant differences between genetic materials, with Moscato Tardío exhibiting higher values (217.32 mg GAE·kg^−1^) compared to Pavía Canario (171.44 mg GAE·kg^−1^) at harvest (Figure 4A). In Pavía Canario, a significant increase was observed after 20 days of refrigerated storage; however, this increase was not sustained over time, as the value measured at 30 days returned to levels like those initially recorded. During shelf life, no significant variations were found, with values ranging from 84.96 to 177.6 mg GAE·kg^−1^. In contrast, Moscato Tardío showed a delayed but marked increase in TPC after 30 days of storage, reaching values 50% higher than at previous times. However, during the shelf life, a significant decrease was observed at 30 + 5 days, with the contents dropping to half. Notably, the most important differences between varieties occurred at 20 days, where Pavía Canario exceeded Moscato Tardío by ~50%, and at 30 days, when Moscato Tardío showed more than double the content of Pavía. During shelf life (except at 30 + 5), Pavía Canario consistently presented at least twice TPC of Moscato Tardío.

Previous research supports these contrasting observations as mentioned by Tsantili et al. [35] that reported decreases in TPC during storage in ‘Harrow Diamond’ peaches kept at 5 °C for 2 and 4 weeks. Conversely, Zhao et al. [36] observed an initial decrease followed by an increase in polyphenol content during the refrigerated storage of peaches at −1, 0, and 5 °C for 28 days, consistent with the dynamic trend found in this study. Such patterns can be explained by the oxidative regulation mechanisms in plant tissues, which involve a dynamic balance between the production and scavenging of ROS. An initial tissue response to stress leads to increased ROS production, which subsequently triggers the activation of antioxidant defense systems [35,37,38]. In our study, the lowest levels of TPC, recorded during shelf life, suggest a diminished antioxidant response capacity, making fruits more vulnerable to oxidative damage. This coincides with sensory evaluations showing higher mealiness and lower juiciness at those times, especially in Pavía Canario, which exhibited the lowest polyphenol content at 8 + 5, 16 + 5, and 20 + 5 days.

#### 2.5.2. Total Antioxidant Capacity (FRAP, DPPH and ABTS)

The total antioxidant capacity (TAC) measured by FRAP also showed significant differences between genetic materials. Pavía Canario recorded a higher average value (117.17 mg AAE·kg^−1^) than Moscato Tardío (95.03 mg AAE·kg^−1^) at harvest (Figure 4B). In Pavía Canario, a marked increase was observed at 8 and 16 days of refrigerated storage, reaching values over 150 mg AAE·kg^−1^. These values decreased slightly afterward but remained higher than those observed at harvest. During shelf life, the values stayed consistently high. Moscato Tardío, on the other hand, maintained nearly constant FRAP values until 20 days of storage. A sharp increase was observed at 30 days, nearly doubling the antioxidant capacity. During the shelf life, values increased again at 16 + 5 and 20 + 5 days but returned to baseline by 30 + 5 days.

In the case of DPPH, Pavía Canario again showed the highest TAC at harvest (472.84 mg AAE·kg^−1^), significantly exceeding Moscato Tardío (389.94 mg AAE·kg^−1^). In most refrigerated storage times, values in Pavía Canario were between 50% and 100% higher (Figure 4C). However, at 20 + 5 days of shelf life, Moscato Tardío presented a value 45% higher than Pavía. Over time, Pavía Canario showed a slight decrease in DPPH up to 8 days and a ~20% increase by day 30. Moscato Tardío, in contrast, exhibited a decreasing trend during storage, followed by a late increase during shelf life (particularly at 20 + 5 days).

As for the ABTS assay, no significant differences were observed between varieties, though relevant intra-varietal variations were detected during storage (Figure 4D). In Pavía Canario, a substantial increase occurred after 20 and 30 days of cold storage, with values nearly tripling those at previous time points. These values remained high during shelf life (between 2148.02 and 2817.25 mg AAE·kg^−1^). In Moscato Tardío, maximum values were recorded at 8 and 20 days and remained relatively stable throughout shelf life (773.11–2756.66 mg AAE·kg^−1^), albeit with greater variability.

The responses observed in TAC reinforce the idea that exposure to low temperatures may stimulate the biosynthesis of antioxidant compounds as a defense mechanism. In nectarines, an increase in flavonoid content after 20 days of storage at 4 ± 1 °C was reported, suggesting this may represent an adaptive response to low-temperature stress. According to those authors, plant tissues under stress generate free radicals that trigger antioxidant system activation to stabilize cellular structures [38].

Together, these results highlight the complex and genotype-dependent nature of antioxidant responses, with Pavía Canario showing a more immediate activation of defenses and Moscato Tardío exhibiting delayed but significant responses. Differences among antioxidant assays also reflect their sensitivity to distinct compounds and mechanisms within the antioxidant network.

### 2.6. Individual Polyphenols

The polyphenols determined are presented in Table 2 and Table 3. The most significant variations during refrigerated storage were observed in catechin. At harvest, values ranged between 52.69 and 44.41 mg·kg^−1^ FW for Pavía Canario and Moscato tardío, respectively. After 8 days, catechin levels in Pavía Canario were 2.5 times higher than those in Moscato tardío. However, by the end of refrigerated storage, Moscato tardío exhibited nearly twice the catechin content compared to Pavía Canario. This dynamic change may reflect varietal differences in the synthesis and degradation pathways of catechin during storage, potentially influenced by their distinct genetic backgrounds and metabolic responses to cold stress [39,40].

During shelf life, differences between varieties were evident at 8 + 5 days, with Pavía Canario presenting 40% less catechin than Moscato tardío. Otherwise, catechin levels remained largely stable in both varieties throughout shelf life, suggesting that post-refrigeration conditions exert a limited impact on this compound’s stability. Catechin is recognized for its antioxidant properties, which contribute to the fruit’s defense against oxidative damage and may potentially influence sensory attributes such as bitterness and astringency, thereby affecting consumer acceptance [41,42].

Moscato tardío contained 10% more chlorogenic acid than Pavía Canario. Differences between varieties were observed at 8 days when a reduction occurred in Moscato tardío, and at 30 days of storage when Pavía Canario showed a decrease. During shelf life, at 20 + 5 days, the chlorogenic acid content in Pavía Canario decreased by 40% compared to Moscato tardío, whose levels remained practically unchanged. Chlorogenic acid is one of the major phenolics in peaches and plays a critical role in antioxidant capacity and browning reactions. The observed variation suggests differential enzymatic activities, such as polyphenol oxidase, between the two varieties, which could impact postharvest quality and shelf life [41].

The *p*-coumaric acid content in Pavía Canario at 8, 16, and 20 days exceeded that of Moscato tardío by approximately 5%, with Moscato tardío maintaining stable levels throughout refrigerated storage. In Pavía Canario, no significant variations were detected until 20 days, but by 30 days, a 7% reduction was observed. During shelf life, Moscato tardío maintained stable levels. In contrast, Pavía Canario—despite having higher content than Moscato tardío at 8 + 5 and 16 + 5 days—showed reductions of 7% and 10%, respectively, compared to values measured at the end of refrigerated storage. *p*-coumaric acid is involved in plant defense mechanisms and may influence flavor and antioxidant properties, so its decrease could imply a reduction in these beneficial traits over time [43].

Quercetin and rutin contents exhibited similar behavior, remaining practically constant during storage and shelf life. Average values for Pavía Canario were 1.91 and 1.23 mg·kg^−1^ FW, and for Moscato tardío, 1.81 and 1.20 mg·kg^−1^ FW for quercetin and rutin, respectively. Cyanidin levels differed between varieties, with 116.33 mg·kg^−1^ FW in Pavía Canario and 94.95 mg·kg^−1^ FW in Moscato tardío. As a primary anthocyanin pigment, cyanidin contributes to fruit color and antioxidant capacity, which are key quality parameters influencing marketability. The stability of these flavonoids suggests resilience to storage conditions and potential sustained health benefits throughout the postharvest [39]. Ferulic and gallic acid contents were 2.89 and 10.91 mg·kg^−1^ FW in Pavía Canario and 2.81 and 8.95 mg·kg^−1^ FW in Moscato tardío, respectively. Caffeic acid showed the most pronounced difference between varieties, with levels ten times higher in Moscato tardío (14.08 mg·kg^−1^ FW) compared to Pavía Canario (1.4 mg·kg^−1^ FW). The marked disparity in caffeic acid could be linked to varietal metabolic pathways and may influence antioxidant activity and potential health-promoting properties [42,43,44].

Both the phenolic compounds and their measured contents agree with those reported by other authors. For example, Zhao et al. [45] evaluated five flat peach cultivars and identified nine phenolic compounds, including p-coumaric acid, procyanidin B, neochlorogenic acid, catechin, vanillic acid, chlorogenic acid, epicatechin, rutin, and quercetin. According to these authors, chlorogenic acid was the predominant compound, as was also observed in our study, highlighting the significant variation between genetic materials, with contents ranging from 0.15 to 11.85 mg per 100 g of fresh pulp. This intervarietal variability highlights the importance of selecting cultivars that balance both nutritional quality and postharvest performance [45,46].

### 2.7. Sensory Evaluation

**Figure 5:**
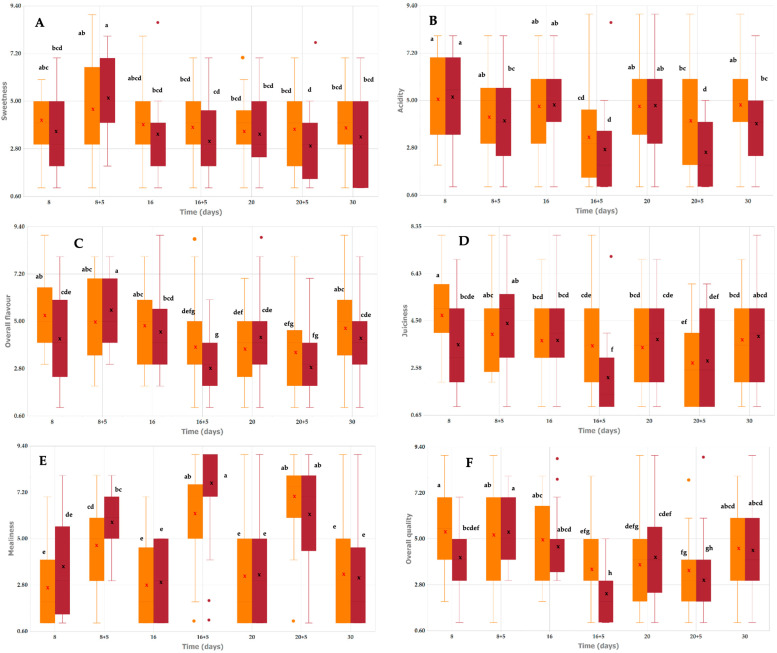
Swetness (**A**), Acidity (**B**), Overall flavour (**C**), Juiciness (**D**), Mealiness (**E**) and Overall quality (**F**) of Pavía Canario and Moscato tardío peach fruits. Values are expressed as median (interquartile range). The central line inside the box represents the median, the cross (x) indicates the mean, and colored circles represent outliers. Different letters within the same row indicate significant differences according to the Kruskal–Wallis test followed by Bonferroni post hoc test (*p* < 0.05).

Sweetness and overall flavour followed a similar pattern: both varieties showed moderate ratings (around 4–5) for most storage times (Figure 5A,C). At 8 + 5 days, values increased noticeably, particularly in Pavía Canario, which reached close to 6, indicating an optimal flavour for consumption. After 16 days, overall flavour decreased, with the lowest ratings observed at 16 + 5 days in Pavía Canario. Acidity showed some fluctuations during storage (Figure 5B). A marked decrease was already perceived at 16 + 5 days, especially in Pavía Canario, and again at 20 + 5 days. Despite a partial recovery at 20 days, values tended to be lower during shelf life periods, with the lowest median acidity recorded in Pavía Canario at 16 + 5 days.

Juiciness varied during storage. At 8 days, Moscato tardío was rated as significantly juicier than Pavía Canario (Figure 5D). From 8 + 5 to 16 days, both varieties maintained moderate scores. A pronounced decrease was observed at 16 + 5 days in Pavía Canario, which reached the lowest juiciness ratings, while Moscato remained stable. During the following storage periods (20 to 30 days), juiciness values were moderate again, without apparent differences between varieties. Mealiness was generally perceived as low in Moscato tardío throughout refrigerated storage (scores around 3–4). In contrast, Pavía Canario showed a progressive increase from 8 + 5 days onwards, reaching its highest values after 16 + 5 and 20 + 5 days (around 7–8 on the 9-point scale). At the end of storage (30 days), both varieties returned to moderate mealiness levels (Figure 5E).

In our study, peaches stored under refrigerated conditions showed variations in sweetness, juiciness, and mealiness over time, with Pavía Canario exhibiting a pronounced decrease in juiciness and an increase in mealiness during later storage periods, while Moscato remained relatively stable. These findings align with previous research [29,47]. In a study by Biacheti et al. [48]. various aspects related to the quality of 52 peach cultivars were analyzed over eight seasons. Sensory evaluation indicated that flavor, particularly sweetness, was the most important attribute driving quality perception, while texture, aroma, and appearance played secondary roles. Panelists also highlighted juiciness as a key factor influencing the eating experience, with firmness and juiciness being the textural attributes most affected by storage time, emphasizing their relevance for peach acceptance and breeding goals. Serra et al. [49] further reported that sweetness, aroma, and juiciness are positively correlated and strongly associated with overall consumer liking. Additionally, cold storage can affect sensory traits such as acidity, sweetness, and aroma, as shown by Christofides et al. [50], who found that nectarines stored at lower temperatures were perceived as more acidic and less sweet than freshly harvested fruit, highlighting the influence of intrinsic fruit attributes over panelist-related factors in determining overall acceptance.

**Figure 6:**
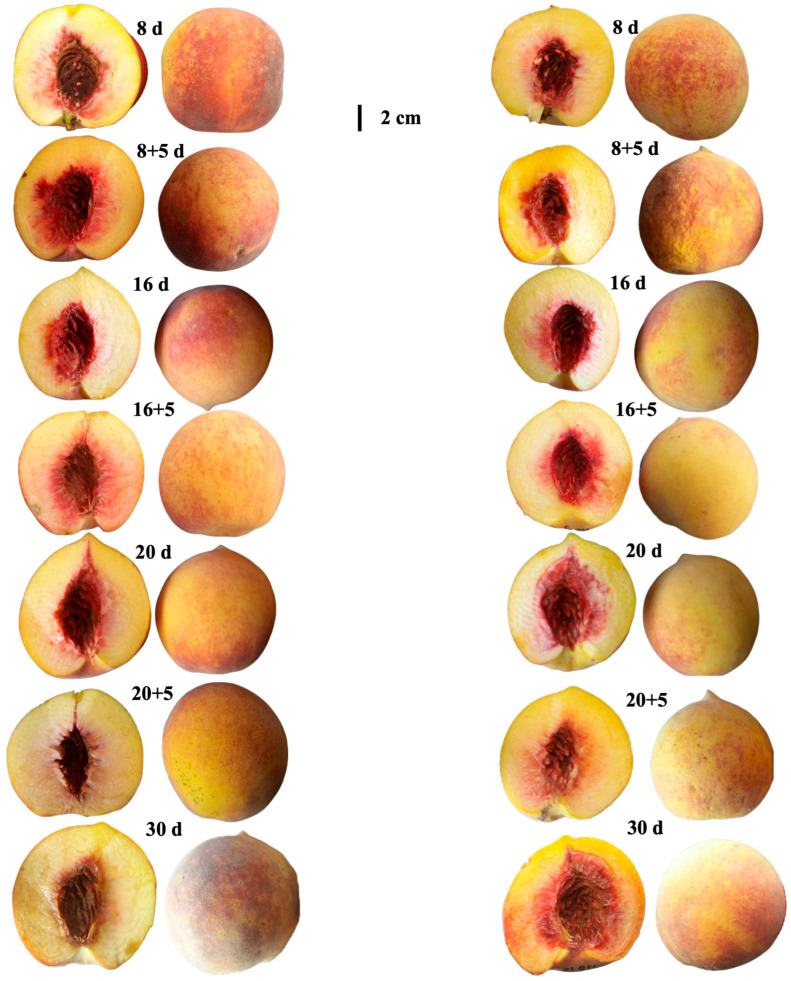
Visual appearance of Pavía Canario (**left**) and Moscato Tardío (**right**) peaches at different storage times and during shelf life.

Regarding overall quality, both varieties were considered acceptable for consumption during refrigerated storage and up to 8 + 5 days of shelf life, when the highest scores were recorded. At 16 + 5 and 20 + 5 days; however, fruits of both varieties were rated as unacceptable, with Pavía Canario showing the steepest decline. By the end of storage (30 days), a partial recovery was observed, although scores remained lower than the initial values (Figure 5F). The visual appareance of the peach were showed on Figure 6.

The sensory evaluation highlighted the very limited commercial shelf life of both peach varieties, with acceptable quality maintained only up to 8 + 5 days. Beyond this point, and particularly after 16 days of refrigerated storage followed by shelf life, fruits showed a marked decline in sweetness, juiciness, and overall quality, together with a pronounced increase in mealiness, leading to ratings considered unacceptable. As expected, the most severe deterioration was observed during shelf life. Between the two varieties, Moscato exhibited greater stability across attributes and higher overall scores, whereas Pavía Canario was more susceptible to mealiness and experienced a sharper decline in sensory quality.

### 2.8. Principal Component Analysis of Biochemical and Sensory Attributes

**Figure 7:**
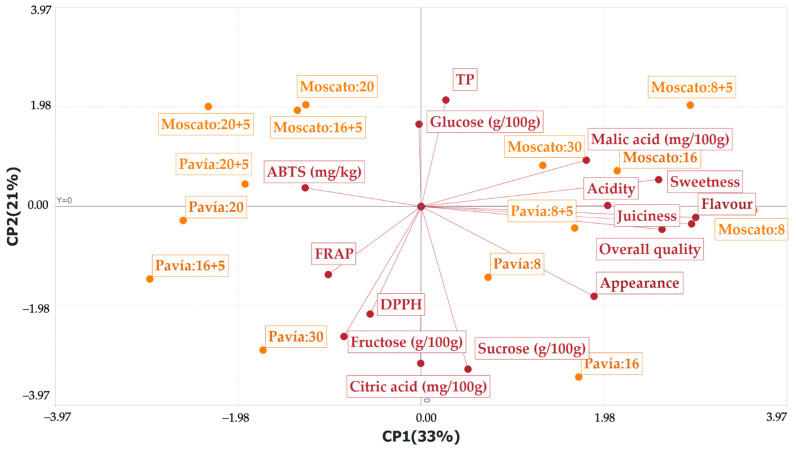
Principal component analysis (PCA) of biochemical and sensory attributes of Moscato Tardío and Pavía Canario peaches during cold storage.

Principal component analysis (PCA) showed that the first two components explained 54% of the total variability, with PC1 contributing 33% and PC2 21% (Figure 7). Considering PC3 (12%) and PC4 (9%) increased the cumulative variance to 75%, providing a more complete representation of the dataset. PC1 (L1) was strongly influenced by positive sensory attributes, such as flavor (0.44), overall quality (0.43), juiciness (0.39), sweetness (0.38), acidity (0.30), and appearance (0.28), as well as by malic acid (0.26). In contrast, fructose (−0.12), ABTS (−0.19), FRAP (−0.15), DPPH (−0.08), and glucose (−0.00) showed negative associations with this component. PC2 (L2) was positively related to total polyphenols (TP, 0.31) and glucose (0.24), whereas sucrose (−0.48), citric acid (−0.46), and fructose (−0.38) loaded negatively. Some sensory attributes had loadings close to zero (juiciness −0.07, flavor −0.03, overall quality −0.05).

Regarding sample distribution, Moscato fruits stored for 8, 30, and 8 + 5 days, as well as Pavía 8 + 5, were located in the positive region of PC1, being associated with greater juiciness, flavor, and overall quality. In contrast, Pavía fruits stored for 16 + 5 and 30 days appeared in the negative region of PC1, showing higher FRAP, DPPH, and fructose values. Pavía fruits stored for 20 and 20 + 5 days exhibited higher antioxidant capacity as measured by ABTS, but lower juiciness and sweetness. Moscato fruits stored for 16 + 5 and 20 days were positioned near the PCA origin, showing intermediate values in both sensory attributes and antioxidant capacity. Overall, the PCA differentiated the two varieties, with Moscato characterized by positive sensory attributes and Pavía associated more with antioxidant capacity, albeit with lower sensory quality. These results allow a clear characterization of Moscato Tardío and Pavía Canario peaches, which can guide the selection of cultivars for direct consumption or processing based on their post-harvest performance.

Other studies have reported that peach cultivars can be grouped based on their chemical composition and sensory attributes [51]. Similarly, our PCA results showed clear separation of Moscato Tardío and Pavía Canario fruits according to their sensory quality and antioxidant content, highlighting cultivar-dependent differences in post-harvest characteristics. In line with this, another study analyzing 212 germplasms found differences in acids, appearance, and sugar–acid ratio, which allowed preliminary selection of superior genetic materials and supported breeding programs [47].

## 3. Materials and Methods

### 3.1. Plant Material

Fruits used in this study were harvested at physiological maturity, which corresponded to a yellow-green skin coloration, pulp firmness values of 25–30 °N, and total soluble solids content of 12–14 °Brix. The peaches were obtained from an 8-year-old orchard located in Melilla, Montevideo, Uruguay (−34.812° S, −56.1636° W). Harvesting was performed manually at the end of February and the beginning of March. Fruits were collected from approximately 20 trees, from the middle portion of each tree, and placed in bags before transferring them into 25 kg plastic boxes. After harvest, they were transported (about 45 km) to the Laboratorio Poscosecha de frutas y hortalizas on the Facultad de Agronomía (Montevideo, Uruguay). Fruits were selected to be representative of each cultivar, based on uniform size, absence of defects, and commercial maturity, and stored in disassemblable corrugated plastic boxes (three of 21 fruit for each analysis moment), at 0 °C and 98% RH for 30 days. The analysis was carried out after harvest, 8, 16, 20, and 30 days of cold storage. After each storage period, fruits were placed in shelf life condition (20 °C and about 85% RH) for 5 days.

### 3.2. Respiration Rate

The respiration rate was determined by the stationary method. Five fruits were placed in a hermetically sealed glass jar of 1 L with a plastic lid with a silicon septum on the top to allow headspace gas sample extraction. On evaluation days, jars were sealed for one hour. After that, gaseous samples, extracted with a discardable syringe of the headspace, were injected on a gas chromatograph (Agilent, 7890b, Santa Clara, CA, USA) with a flame ionization detector (FID) and an 80/100 packed column (Agilent, HayeSep Q, Santa Clara, CA, USA) of 3.66 m long and 2 mm internal diameter, to determine CO_2_ production. The carrier gas was high-purity nitrogen (Linde, Montevideo, Uruguay), and the injector, detector, and oven temperatures were 100, 300, and 60 °C, respectively. A CO_2_ standard with a concentration of 600 ppm was used (Agilent, Santa Clara, CA, USA).

### 3.3. Flesh Firmness Measurment

Flesh firmness was determined on two opposite sides (exposed and shaded) of the equatorial zone, including the suture and shoulder regions of the fruit. Measurements were performed by penetrometry using a TA.XT Plus Express texture analyzer (Stable Microsystems, London, UK) equipped with a 7 mm diameter cylindrical probe (P 7/D). For each variety and storage time, three independent replicates were analyzed, each consisting of 21 fruits (7 fruits from each of the three plastic boxes) measured individually. The peel was removed prior to measurement, and values were expressed in N.

### 3.4. Weight Loss Determination

To determine weight loss, the fruits were weighed using an analytical balance (Acculab VI, 1000 ± 1 g, Central Islip, NY, USA) at the time of harvest and at each time of shipment. The same replication scheme described for firmness was applied. Weight loss was calculated as the difference between the initial and final weight and the values were expressed as a percentage.

### 3.5. Total Solid Soluble Contents, Organic Acid and Sugar Determination

Total soluble solids (TSS) were determined by extracting a drop of juice from each fruit and measuring it using a handheld digital refractometer (Atago 3810 PAL-1, range 0.0–53.0%, Inagi, Tokio, Japan). The same replication scheme used for flesh firmness and weight loss was also applied, with each replicate consisting of 21 fruits (7 fruits from each of the three plastic boxes) measured individually.Values were expressed in °Brix.

The determination of organic acids and sugar was carried out by high-performance liquid chromatography (HPLC) using a Shimadzu HPLC system (SIL-20AC autosampler; Shimadzu Corporation, Kyoto, Japan) equipped with a diode array detector (DAD-20A).The juice obtained by pooling a piece from each of the 21 fruits in a replicate was filtered through 0.45 μm nylon membrane filters (Merck Millipore, Darmstadt, Germany), and injected into the HPLC system. Separation was performed using a Shodex SH1821 column (6 µm, 8.0 mm internal diameter × 300 mm long; Shodex, Tokyo, Japan). The mobile phase consisted of 0.01 N H_2_SO_4_, delivered at a flow rate of 1 mL/min. The column oven was maintained at 50 °C, and the total run time was 25 min. Three independent replicates were analyzed for each variety and storage time. Values were expressed as mg per 100 g fresh weight (FW).

### 3.6. Total Polyphenol Contents and Total Antioxidant Capacity Determination

Prior to analysis, a composite sample was prepared by cutting a portion of pulp from each of the 7 fruits in a replicate, homogenizing them, and taking 1 g of the resulting mixture. This sample was further homogenized with 3 mL of methanol–water solution (70:30 *v*/*v*) using a Labotech Science homogenizer (XHF-D, Beijing, China) and centrifuged at 15,000× *g* for 10 min at 4 °C (Thermo Scientific, Sorval ST 16R, Osterode am Harz, Germany). Total phenolic content (TPC) was determined according to Singleton & Rossi [52] using 19.2 μL of the extract that reacted with 29 μL of diluted Folin’s reagent (1:1 *v/v* with distilled water) and 192 μL of a solution containing 0.4% NaOH (Carlo Erba Reagent, Milan, Italy) and 2% sodium carbonate (Merck KGaA, Darmstadt, Germany). The mixture was incubated in microplates at 20 °C for 1 h, and absorbance was measured at 765 nm using a plate spectrophotometer (Multiskan Sky, Thermo Fisher Scientific, Madison, WI, USA). Gallic acid (Merck KGaA, Darmstadt, Germany) was used for the calibration curve, and results were expressed as mg gallic acid equivalents (mg GAE) per 100 g fresh weight (FW). Three independent replicates were analyzed for each variety and storage time.

The same composite sample prepared for total phenolic content was used as the substrate for subsequent antioxidant and enzyme activity assays. For each assay, an aliquot of the extract obtained from this sample was used, following the specific protocols described below. Total antioxidant capacity (TAC) was evaluated using the ferric reducing antioxidant power (FRAP), 2,2-diphenyl-1-picrylhydrazyl (DPPH) assay y 2,2′-azino-bis(3-ethylbenzothiazoline-6-sulfonic acid) (ABTS) assay. The FRAP assay was performed according to Benzie & Strain [53] using 10 μL of extract were mixed with 190 μL of FRAP reagent in microplates, covered to protect from light, and incubated at room temperature for 45 min. Absorbance was measured at 593 nm. DPPH assay was performed using a stock DPPH solution that was prepared and adjusted with ethanol to 1.1 [54]. Then, 10 μL of extract were mixed with 190 μL of diluted DPPH solution, incubated in the dark at room temperature for 30 min, and absorbance was measured at 515 nm. For ABTS assay, an ABTS radical cation solution was prepared by mixing 7 mM ABTS with 2.45 mM potassium persulfate in 10 mL distilled water and incubating in the dark at room temperature for 12–16 h. For the assay, 10 μL of extract were added to 190 μL of the diluted ABTS solution in microplates, incubated in the dark at room temperature for 30 min, and absorbance was measured at 734 nm.

Calibration curves were constructed using Trolox (Merck KGaA, Darmstadt, Germany) for all TAC assays. Results were expressed as mg Trolox equivalents per 100 g FW.

### 3.7. Individual Polyphenols Content

For individual phenolic compound analysis, an aliquot of the same extract prepared for total phenolic content was used, following the procedure described below. The extract was filtered through 0.45 μm nylon membrane filters (Merck Millipore, Darmstadt, Germany) and injected into a himadzu HPLC system (SIL-20AC autosampler; Shimadzu Corporation, Kyoto, Japan) equipped with a diode array detector (DAD-20A). Separation was achieved using a Restek C18 column (5 μm, 250 × 6 mm; Restek, Bellefonte, PA, USA). The mobile phase consisted of 1% formic acid in water (A) and 100% acetonitrile (B), delivered at a flow rate of 0.9 mL/min. The column oven was maintained at 35 °C, and the total run time was 35 min. The injection volume was 10 μL. Three independent replicates, corresponding to the same composite samples used for total phenolic content, were analyzed for each variety and storage time. Results were expressed in mg per 100 g fresh weight (FW).

### 3.8. Sensory Evaluation

The variables appearance, sweetness, acidity, flavor, mealiness, juiciness, and overall acceptance were evaluated using a 9-point hedonic scale. A score of 1 corresponded to the lowest rating (very bad for appearance and flavor; not sweet or acidic; not juicy; very mealy; and very disliked for overall acceptance), and a score of 9 corresponded to the highest rating (very good for appearance and flavor; very sweet or acidic; juicy; not mealy; and very liked for overall acceptance). A score of 5 was considered acceptable for all variables.

A total of 32 panelists (approximately 60% female and 40% male, aged 20–60 years) participated in the evaluation. Samples were prepared immediately before serving, presenting each fruit whole along with two slices. Samples were distributed in a random order. Panelists were instructed to rinse their mouths with mineral water between samples to avoid fatigue effects.

### 3.9. Principal Component Analysis

Relationships between biochemical and sensory attributes were analyzed by Principal Component Analysis (PCA). Variables included in the PCA were: sugars (glucose, fructose, sucrose), organic acids (citric and malic acid), total polyphenols (TP), antioxidant capacity (ABTS, FRAP, DPPH), and sensory attributes (flavor, sweetness, acidity, juiciness, overall quality, appearance). Biplots were generated to visualize sample distribution and variable loadings.

### 3.10. Statistical Analysis

Statistical analyses were performed using Navure software (v. 2.7.3) with R version 3.6.3 [55]. A factorial ANOVA was applied to evaluate the effect of two factors, variety (Pavía Canario and Moscato tardío) and storage time (harvest, 8, 16, 20, and 30 days of storage and the corresponding 7-day shelf life), as well as their interactions. When significant differences were detected (*p* < 0.05), Tukey’s multiple comparisons test was used to determine differences between levels within each factor. Results are presented as mean ± standard error of the mean.

Sensory data did not meet the assumptions of normality (Shapiro–Wilk test, *p* < 0.05) or homoscedasticity (Levene’s test, *p* < 0.05). Therefore, a non-parametric Kruskal–Wallis test was applied to evaluate the effect of storage time and variety on each sensory attribute, followed by pairwise comparisons with Bonferroni adjustment when significant differences were found. The number of replicates varied depending on the variable and is indicated in the corresponding sections.

## 4. Conclusions

Overall, the postharvest responses of the two peach varieties were clearly genotype-dependent. Moscato Tardío exhibited a sustained pattern of cold tolerance, characterized by moderate-to-high respiratory activity, firmer texture during refrigerated storage, delayed but substantial increases in total polyphenols and antioxidant capacity, and higher glucose and sucrose content. The higher respiration observed in Moscato Tardío may reflect higher initial stress due to the cold. However, it could also provide additional energy to activate antioxidant defenses and cellular repair mechanisms that could mitigate chilling stress effects. These traits likely contributed to the better maintenance of sensory quality, particularly juiciness and reduced mealiness, throughout storage and shelf life. In contrast, Pavía Canario showed an earlier activation of antioxidant defenses and higher initial sucrose levels, but experienced more pronounced juice loss, mealiness, and fluctuations in polyphenol content during shelf life, indicating higher susceptibility to chilling injury. The evolution of firmness, loss of juiciness, weight changes, and antioxidant responses demonstrates that chilling injury is multifactorial, depending on the balance between metabolic stress, tissue structure, and the antioxidant capacity of each variety.

## Figures and Tables

**Table 1:** Organic acid (citric and malic) and sugars (sucrose, glucose and fructose) in Pavía Canario and Moscato tardío peaches during storage and shelf life.

Time of Storage and Shelf Life	Malic Acid (mg 100 g^−1^ FW)		Citric Acid (mg 100 g^−1^ FW)		Sucrose (g 100 g^−1^ FW)		Fructose (g 100 g^−1^ FW)		Glucose (g 100 g^−1^ FW)	
	P. Canario	M. Tardío	P. Canario	M. Tardío	P. Canario	M. Tardío	P. Canario	M. Tardío	P. Canario	M. Tardío
**Harvest**	568.33 ± 18.03 Aa	586.49 ± 21.83 Aa	144.01 ± 5.36 Aa	89.93 ± 4.63 Ba	1.81± 0.81 c	2.45 ± 0.12 a	1.14 ± 0.32	0.77 ± 0.02	1.17 ± 0.02 Bb	1.41 ± 0.10 Ab
**8 d**	356.76 ± 14.44 Babc	420.67 ± 8.43 Acd	71.02 ± 3.54 Acd	84.76 ± 3.97 Aab	2.42 ± 0.11 abc	2.17 ± 0.04 abc	0.95 ± 0.05	0.76 ± 0.04	1.02 ± 0.05 Aab	1.24 ± 0.07 Ab
**16 d**	428.01± 11.28 Aa	402.82 ± 26.18 Acd	126.74 ± 1.91 Aa	65.83 ± 1.62 Babc	2.74 ± 0.25 a	2.37 ± 0.09 ab	1.02 ± 0.08	0.61 ± 0.02	1.24 ± 0.09 Bab	1.56 ± 0.08 Aab
**20 d**	329.03 ± 6.53 Bbc	390.88 ± 11.65 Acd	67.81 ± 2.67 Acd	56.39 ± 0.35 Abc	2.23 ± 0.06 abc	2.04 ± 0.02 bcd	0.89 ± 0.02	0.76 ± 0.01	1.07 ± 0.06 Bab	1.69 ± 0.06 Aab
**30 d**	289.68 ± 5.37 Bcd	397.42 ± 8.61 Acd	120.87 ± 1.49 Aab	54.67 ± 5.58 Bc	2.38 ± 0.11 abc	2.01 ± 0.11 bcd	0.86 ± 0.05	0.95 ± 0.09	1.15 ± 0.09 Bab	1.88 ± 0.09 Aa
**8 + 5 d**	334.69 ± 19.52 Abc	374.74 ± 23.46 Acd	56.56 ± 3.73 Ad	54.16 ± 3.32 Ac	2.26 ± 0.10 abc	1.76 ± 0.03 de	0.72 ± 0.03	0.62 ± 0.03	0.94 ± 0.04 Bab	1.25 ± 0.07 Ab
**16 + 5 d**	315.54 ± 25.23 Abc	348.70 ± 15.78 Ac	73.86 ± 9.98 Acd	54.85 ± 4.62 Bc	2.52 ± 0.10 ab	1.74 ± 0.03 de	1.02 ± 0.03	0.58 ± 0.02	1.41 ± 0.04 Aab	1.32 ± 0.04 Ab
**20 + 5 d**	386.41 ± 11.27 Aab	376.04 ± 11.93 Acd	94.33 ± 5.77 Abc	49.33 ± 1.76 Bc	1.84 ± 0.08 bc	1.51 ± 0.02 e	0.93 ± 0.02	0.71 ± 0.01	1.29 ± 0.06 Aab	1.36 ± 0.06 Ab
**30 + 5 d**	230.67 ± 4.24 Bd	425.57 ± 16.01 Ab	93.52 ± 5.10 Abc	56.33 ± 8.16 Bbc	1.78 ± 0.11 c	1.81 ± 0.14 cde	0.91 ± 0.07	0.85 ± 0.03	1.42 ± 0.07 Aa	1.42 ± 0.13 Aab
***p* (variety × storage time)**	<0.001		<0.001		0.8385		NS		<0.001	
***p* variety**	<0.001		<0.001		0.0017		<0.001		<0.001	
***p* time**	<0.001		<0.001		0.0010		NS		<0.001	

Values are means (n = 3) ± standard error of the mean. Uppercase letters indicate differences between varieties at each evaluation day, and lowercase letters indicate differences over time within each variety (Tukey’s test, *p* < 0.05). NS not significant.

**Table 2:** Phenolic compounds in Pavía Canario and Moscato tardío peaches during storage and shelf life.

Time of Storage and Shelf Life	Catechin (mg kg^−1^ FW)		Chlorogenic Acid (mg kg^−1^ FW)		*p*-Coumaric Acid (mg kg^−1^ FW)		Quercetin (mg kg^−1^ FW)	
	P. Canario	M. Tardío	P. Canario	M. Tardío	P. Canario	M. Tardío	P. Canario	M. Tardío
**Harvest**	52.69 ± 1.82 Aab	44.41 ± 2.07 Abc	21.43 ± 1.02 Aab	24.06 ±1.08 Aa	2.94 ± 0.04 Aa	2.89 ± 0.01 Ba	1.95 ± 0.03 Abc	1.84 ± 0.01 Ba
**8 d**	60.69 ± 0.94 Aa	26.26 ± 2.37 Bd	25.03 ± 0.70 Aa	15.85 ± 0.83 Bbc	2.96 ± 0.01 Aa	2.80 ± 0.01 Ba	2.01 ± 0.03 Ac	1.77 ± 0.01 Ba
**16 d**	28.35 ± 1.98 Ade	33.91± 0.17 Abcd	19.47 ± 2.40 Aabc	18.78 ± 0.41 Aabc	2.93 ± 0.05 Ab	2.78 ± 0.03 Ba	1.85 ± 0.04 Acd	1.81 ± 0.02 Aa
**20 d**	45.55 ± 4.84 Aabc	42.77 ± 3.54 Abc	16.96 ± 0.57 Abc	19.36 ± 0.33 Aabc	2.92 ± 0.02 Ab	2.83 ± 0.03 Ba	1.82 ± 0.02 Ad	1.80 ± 0.03 Aa
**30 d**	22.95 ± 1.54 Be	45.66 ± 6.07 Ab	12.43 ± 1.18 Bc	22.73 ± 1.57 Aab	2.74 ± 0.01 Ab	2.79 ± 0.04 Aa	1.83 ± 0.01 Ad	1.79 ± 0.02 Aa
**8 + 5 d**	39.11 ± 0.28 Bbcd	64.43 ± 4.51 Aa	19.69 ± 1.36 Bab	23.06 ± 2.82 Aa	2.96 ± 0.02 Aa	2.82 ± 0.04 Ba	2.16 ± 0.05 Aa	1.83 ± 0.01 Ba
**16 + 5 d**	39.19 ± 2.24 Abcd	43.33 ± 3.35 Abc	20.52 ± 2.14 Aba	22.58 ± 1.42 Aab	2.94 ± 0.01 Aa	2.78 ± 0.02 Ba	1.85 ± 0.01 Acd	1.77 ± 0.02 Ba
**20 + 5 d**	43.85 ± 0.62 Abcd	42.51 ± 2.90 Abc	16.52 ± 1.39 Bbc	25.50 ± 1.47 Aa	2.76 ± 0.01 Bb	2.80 ± 0.03 Aa	1.86 ± 0.01 Acd	1.82 ± 0.01 Aa
**30 + 5 d**	31.26 ± 3.35 Acde	28.98 ± 1.32 Acd	17.49 ± 2.48 Abc	14.93 ± 1.43 Ac	2.68 ± 0.02 Bb	2.80 ± 0.03 Aa	1.82 ± 0.03 Ad	1.81 ± 0.03 Aa
***p* (variety × storage time)**	<0.001		<0.001		<0.001		<0.001	
***p* variety**	0.4886		0.0115		<0.001		<0.001	
***p* time**	<0.001		0.002		<0.001		<0.001	

Values are means (n = 3) ± standard error of the mean. Uppercase letters indicate differences between varieties at each evaluation day, and lowercase letters indicate differences over time within each variety (Tukey’s test, *p* < 0.05).

**Table 3:** Phenolic compounds in Pavía Canario and Moscato tardío peaches during storage and shelf life.

Time of Storage and Shelf Life	Caffeic Acid (mg kg^−1^ FW)		Rutin (mg kg^−1^ FW)		Cyanidin 3-Glucoside (mg kg^−1^ FW)	
	P. Canario	M. Tardío	P. Canario	M. Tardío	P. Canario	M. Tardío
**Harvest**	1.15 ± 0.35	13.60 ± 0.44	1.15 ± 0.08 Bb	1.33 ±0.01 Ab	124.71 ± 11.22 Abc	102.04 ± 5.98 Bb
**8 d**	1.84 ± 0.48	11.30 ± 0.38	1.45 ± 0.01 Aa	1.18 ± 0.04 Bbc	108.22 ± 1.94 Bcd	139.98 ± 12.75 Aa
**16 d**	1.41 ± 0.38	14.42 ± 0.37	1.22 ± 0.07 Aab	1.22 ± 0.09 Abc	141.73 ± 4.97 Ab	80.97 ± 1.85 Bb
**20 d**	1.31 ± 0.39	14.39 ± 0.32	1.19 ± 0.01 Aab	1.06 ± 0.03 Bc	88.24 ± 1.76 Ad	80.75 ± 0.54 Ba
**30 d**	1.17 ± 0.35	13.85 ± 0.34	1.08 ± 0.03 Ab	0.98 ± 0.01 Ac	190.30 ± 5.59 Aa	79.70 ± 2.17 Bb
**8 + 5 d**	1.44 ± 0.41	18.01 ± 0.40	1.27 ± 0.02 Aab	1.30 ± 0.08 Ab	91.16 ± 2.95 Ad	91.87 ± 0.64 Ab
**16 + 5 d**	1.67 ± 0.42	15.19 ± 0.42	1.33 ± 0.06 Aab	1.33 ± 0.06 Ab	97.77 ± 3.22 Acd	111.51 ± 12.58 Aab
**20 + 5 d**	1.37 ± 0.37	14.57 ± 0.33	1.18 ± 0.05 Ab	1.01 ± 0.02 Bc	90.85 ± 1.10 Ad	82.26 ± 1.14 Ab
**30 + 5 d**	1.27 ± 0.38	11.37 ± 0.55	1.21 ± 0.06 Bab	1.73 ± 0.06 Aa	113.99 ± 3.02 Abcd	85.48 ± 1.71 Bb
***p* (variety × storage time)**	NS		<0.001		<0.001	
***p* variety**	0.0625		0.9878		<0.001	
***p* time**	0.0543		<0.001		<0.001	

Values are means (n = 3) ± standard error of the mean. Uppercase letters indicate differences between varieties at each evaluation day, and lowercase letters indicate differences over time within each variety (Tukey’s test, *p* < 0.05). NS not significant

## Data Availability

The data presented in this study are not publicly available but may be obtained from the corresponding author upon reasonable request.

## Abbreviations

The following abbreviations are used in this manuscript:

ABTS	2,2′-azino-bis(3-ethylbenzothiazoline-6-sulfonic acid)
DAD	Diode array detector
DPPH	2,2-diphenyl-1-picrylhydrazyl
FRAP	Ferric reducing antioxidant power
FW	Fresh weight
GAE	Gallic acid equivalents
HPLC	High-performance liquid chromatography
N	Newton
RID	Refractive index detector
ROS	Reactive oxygen species
TAC	Total antioxidant capacity
TE	Trolox equivalents
TPC	Total polyphenol content
TSS	Total soluble solids
